# Kartagener Syndrome Complicated by Middle and Lower Lobar Mucinous Adenocarcinoma in the Left Lung

**DOI:** 10.5761/atcs.cr.25-00150

**Published:** 2025-10-28

**Authors:** Guang-Yuan Shao, Cheng-De Wang, Dong Wang, Si-Yuan Sun, Bao-Kai Wang, Xiao-Nu Peng, Wen-Quan Yu

**Affiliations:** 1 Department of Thoracic Surgery, The Affiliated Yantai Yuhuangding Hospital of Qingdao University, Yantai, China; 2 Department of General Surgery, The Affiliated Yantai Yuhuangding Hospital of Qingdao University, Yantai, China; 3 Department of Esophageal and Mediastinal Surgery, Shandong Cancer Hospital and Institute, Shandong First Medical University and Shandong Academy of Medical Sciences, Jinan, China

**Keywords:** Kartagener syndrome, mucinous adenocarcinoma, left middle and lower lobe, primary ciliary dyskinesia

## Abstract

**Introduction:**

Kartagener syndrome (KS), a distinct subtype of primary ciliary dyskinesia, is linked to progressive lung disease; concurrent pulmonary mucinous adenocarcinoma mimicking pneumonia is rarely reported and easily misdiagnosed.

**Case Presentation:**

A 64-year-old female presented with years of recurrent cough and sputum. Chest computed tomography (CT) revealed bilateral chronic inflammation, interstitial changes, a left lower lobe mass-like shadow, partial bronchiectasis, and dextrocardia. Bronchoscopy showed chronic mucosal inflammation in the left lower lobe base segment; sputum culture was negative. Symptoms improved with antibiotics/expectorants. Two months later, worsening symptoms prompted re-evaluation. Extensive diagnostic tests (tumor markers, bacteriological/mycological, immunological, viral) were largely negative. CT-guided percutaneous lung biopsy confirmed invasive mucinous adenocarcinoma. Preoperative evaluation revealed situs inversus totalis, chronic sinusitis, and bronchiectasis, confirming concurrent KS. Following multidisciplinary discussion, she underwent thoracoscopic left middle and lower lobectomy with uncomplicated recovery; pathology confirmed R0 resection. She completed 5 cycles of adjuvant pemetrexed/platinum chemotherapy and remains recurrence-free on follow-up.

**Conclusions:**

This represents the first documented case of KS coexisting with pulmonary invasive mucinous adenocarcinoma, to some extent expanding the clinical spectrum of ciliopathy-associated lung malignancies. It suggests that clinicians and radiologists should consider the possibility of concurrent mucinous adenocarcinoma in KS patients.

## Abbreviations

KS
: Kartagener syndrome
PCD
: primary ciliary dyskinesia
IMA
: invasive mucinous adenocarcinoma
CT
: computed tomography
SCCA
: squamous cell carcinoma antigen
CEA
: carcinoembryonic antigen
AFP
: alpha-fetoprotein
CA19-9
: carbohydrate antigen199
ANA
: antinuclear antibody
anti-Sm
: anti-Smith antibodies
anti-SSA
: anti-Sjogren’s syndrome-related antigen A
anti-SSB
: anti-Sjogren’s syndrome-related antigen B
COVID-19
: coronavirus disease 2019
TTF-1
: thyroid transcription factor 1
CK
: cytokeratin
KRAS
: Kirsten rat sarcoma viral oncogene homolog

## Introduction

Kartagener syndrome (KS) is a rare, highly genetically heterogeneous form of motile ciliopathy—a distinct subtype of primary ciliary dyskinesia (PCD)—characterized by the triad of situs inversus totalis, chronic sinusitis, and bronchiectasis. Accounting for approximately 50% of all PCD cases, this highly genetically heterogeneous disorder has an estimated prevalence of <1:10000 live births.^1–3)^ As the second most common inherited airway disorder following cystic fibrosis, PCD causes dysfunctional mucociliary clearance due to impaired ciliary motility, leading to progressive respiratory sequelae, including chronic productive cough, recurrent lower respiratory infections, and irreversible bronchial dilatation.^4)^ Characteristic thoracic imaging findings include atelectasis, bronchial wall thickening, mucus plugging, and/or air trapping and ground-glass opacity^2)^—features that radiologically overlap with those of pulmonary invasive mucinous adenocarcinoma (IMA). IMA is an independent pathological subtype of lung adenocarcinoma, constituting approximately 5% of lung adenocarcinomas.^5)^ It demonstrates unique pathological features characterized by proliferating mucin-secreting glandular epithelium with extracellular mucin accumulation.^6)^ This leads to the chest computed tomography (CT) characteristics resembling inflammatory pulmonary lesions. Due to its lack of specificity, it is easily misdiagnosed as inflammatory nodules, pulmonary tuberculosis, diffuse lesions, or hamartomas, resulting in delayed treatment and poor prognosis. The diagnostic conundrum intensifies when these 2 rare clinical entities coexist. We herein present a surgically managed case of KS complicated by IMA in the left lower and middle lobes, highlighting the importance of multidisciplinary evaluation in such complex presentations. This report aims to enhance diagnostic vigilance among clinicians and radiologists encountering overlapping pulmonary manifestations.

## Case Presentation

**Fig. 1:**
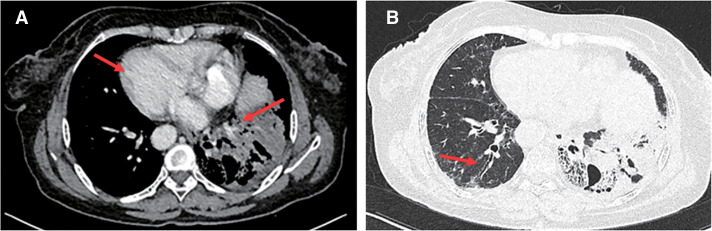
Chest CT (**A**: mediastinal window, **B**: lung window). Chest CT revealed chronic inflammation in both lungs, interstitial changes, a mass-like shadow in the left lower lobe (**A**: the red arrow), partial bronchiectasis (**B**: the red arrow), and dextrocardia (**A**: the red arrow). CT: computed tomography

**Fig. 2:**
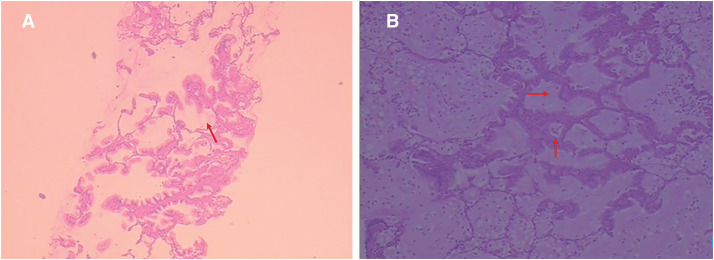
Pathological image. (**A**) Puncture biopsy pathological image and (**B**) postoperative paraffin pathological image, both are consistent with the manifestations of invasive mucinous adenocarcinoma. The labeled section demonstrates neoplastic cells arranged in glandular patterns with mucin pools present in alveolar spaces (the red arrow).

**Fig. 3:**
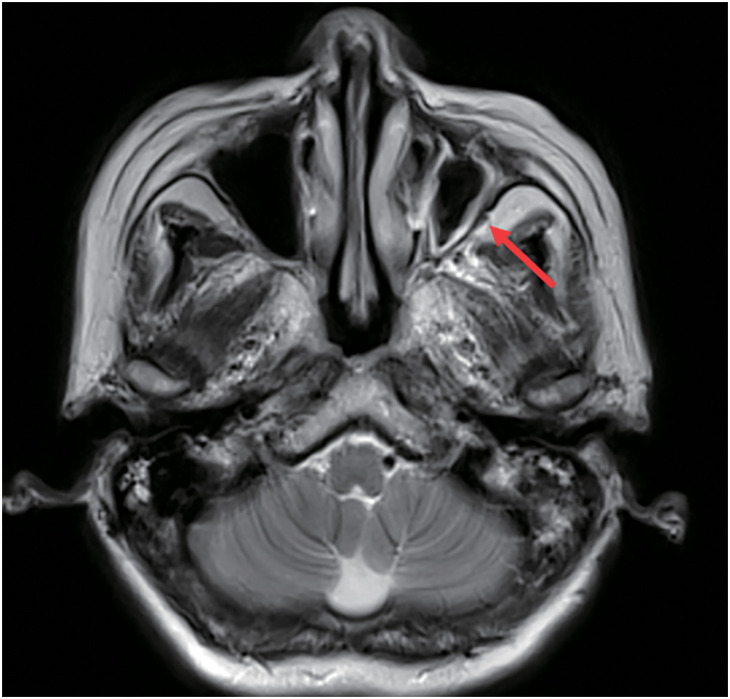
Cranial magnetic resonance imaging. Cranial magnetic resonance imaging demonstrated the presence of sinusitis (the red arrow) in the patient.

**Fig. 4:**
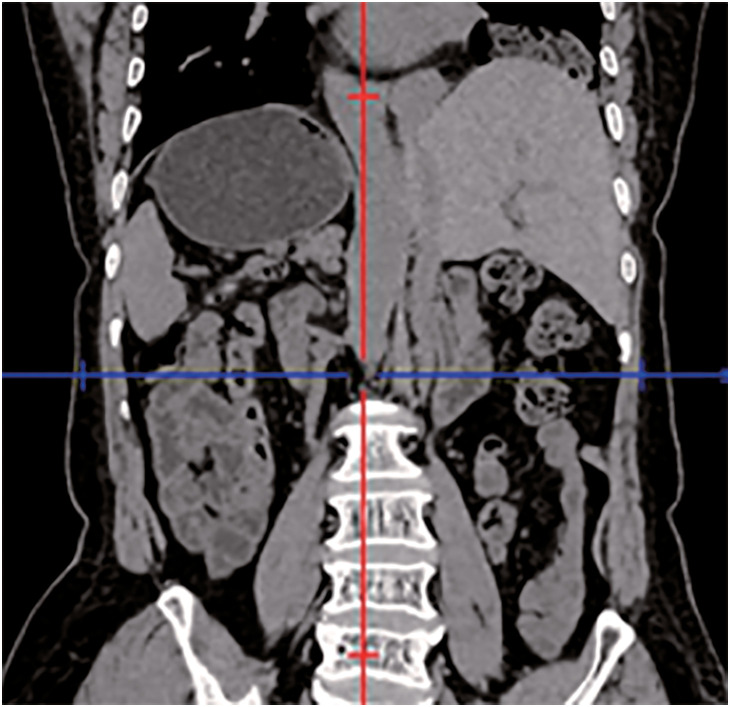
CT of the abdomen (coronal plane). The abdominal CT revealed complete situs inversus totalis, characterized by a mirror-image reversal of organs along the body’s left–right axis: a left-sided liver, right-sided spleen, a predominantly right-sided gastric bubble, and so on. CT: computed tomography

**Fig. 5:**
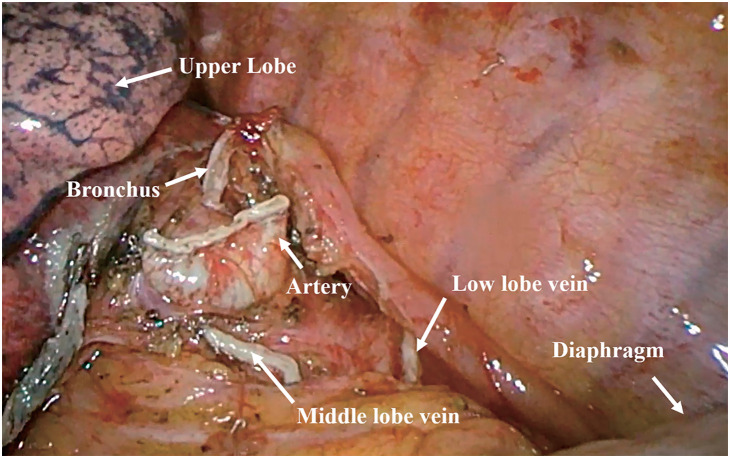
Post-lobectomy thoracoscopy images. The image demonstrates the hilar region following resection of the middle and lower lobes of the left lung, exhibiting a mirror-image change resembling the normal anatomical configuration of the right lung.

**Fig. 6:**
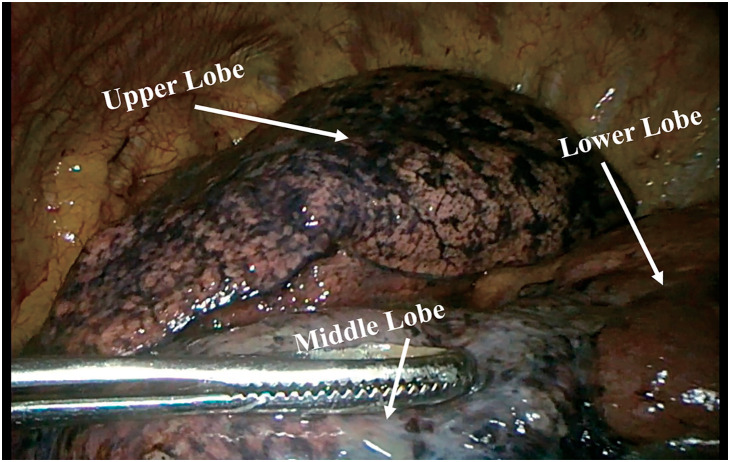
Pre-lobectomy thoracoscopy images. Intraoperative exploration revealed well-developed horizontal and oblique fissures in the left lung.

A 64-year-old female presented to the local hospital due to recurrent coughing and sputum production for several years. There was no known history of confirmed infections prior to the onset of these symptoms, and the patient was a farmer with no history of occupational exposure or smoking. The patient reported no family history of genetically inherited disorders. Chest CT revealed chronic inflammation in both lungs, interstitial changes, a mass-like shadow in the left lower lobe, partial bronchiectasis, and dextrocardia (**Fig. 1**). A bronchoscopy was performed, and the pathology showed chronic mucosal inflammation in the base segment of the left lower lobe. Sputum culture results were negative. After treatment with antibiotics and expectorants, the symptoms improved and the patient was discharged. However, two months later, the symptoms worsened, prompting the patient to seek further evaluation at our hospital. Tumor markers: All were negative except for NSE, which was slightly elevated at 26.3 ng/mL (SCCA, CEA, AFP, CA 19-9, etc.). Autoantibody panels: The antinuclear antibody (ANA) titer was slightly elevated (1:100). No abnormalities were detected in over a dozen other humoral immunity-related markers, including anti-Sm, anti-SSA, and anti-SSB. Sputum bacteriological and fungal cultures again yielded negative results. As it was during the peak of the COVID-19 pandemic in China, viral infections were also ruled out. A follow-up chest CT showed a mass-like shadow in the left lower lobe, with malignancy not excluded, while no significant changes were noted in the other areas. To confirm the diagnosis, the patient underwent a percutaneous lung biopsy. The pathology confirmed a diagnosis of mucinous adenocarcinoma (**Fig. 2**). Immunohistochemistry results were as follows: TTF-1 (−), CK (+), and P40 (−). Upon further evaluation, cranial magnetic resonance imaging demonstrated the presence of sinusitis in the patient (**Fig. 3**). Additionally, an abdominal CT revealed complete situs inversus totalis (**Fig. 4**). These findings collectively indicated a concurrent diagnosis of KS, characterized by the triad of chronic rhinosinusitis, situs inversus, and bronchiectasis. Preoperative evaluation revealed no evidence of distant metastasis or lymph node involvement. Following multidisciplinary team discussion and optimization of pulmonary function to ensure surgical tolerance, the patient underwent thoracoscopic left middle and lower lobectomy in May 2022 (**Fig. 5**). Intraoperative exploration revealed well-developed horizontal and oblique fissures in the left lung (**Fig. 6**). Due to the mirror-reversed situs inversus relative to normal anatomy, the surgical maneuvers were performed in a mirror-reversed manner, posing significant technical challenges. Postoperatively, the patient exhibited increased sputum production compared to typical patients, likely attributed to impaired respiratory mucosal ciliary motility associated with KS. The patient was discharged on the fourth postoperative day following an uneventful recovery. Final pathological examination confirmed mucinous adenocarcinoma without invasion of the visceral pleura (**Fig. 2**). Surgical margins and lymph nodes were negative for tumor involvement, achieving R0 resection. The tumor was staged as pT4N0M0, with Kirsten rat sarcoma viral oncogene homolog (KRAS) mutations positive. The patient completed 5 cycles of postoperative adjuvant chemotherapy with pemetrexed plus platinum-based agents. So far, no evidence of tumor recurrence has been observed during the 3-year outpatient follow-up, and the patient remains currently in good clinical condition.

## Discussion

This report presents a case of KS coexisting with pulmonary mucinous adenocarcinoma. Both conditions share clinical manifestations resembling pneumonia, posing significant diagnostic challenges, particularly when pneumonia is concurrently present.

KS is a rare motile ciliopathy characterized by high genetic heterogeneity.^7)^ As a special subtype of PCD, its diagnosis still requires confirmation through a combination of diagnostic modalities. Current guidelines recommend that PCD can be definitively diagnosed either by transmission electron microscopy demonstrating hallmark ultrastructural defects in cilia or by identification of biallelic pathogenic variants in known PCD-associated genes.^8)^ However, the prohibitive cost of these diagnostic modalities renders them inaccessible in most clinical centers across China. Globally, delayed diagnosis and under-recognition of PCD remain significant challenges.^3,4)^ While clinical manifestations form an essential component of diagnostic evaluation, all PCD-related features stem from abnormal motile ciliary function. Core phenotypes include persistent unexplained respiratory symptoms, laterality defects (situs inversus totalis or situs ambiguus), and chronic wet cough progressing to bronchiectasis, observed in nearly all patients.^2)^ For patients presenting with the classic clinical triad, the diagnosis of KS can be established with relative confidence.^9)^ Nevertheless, in this case, recurrent cough and concomitant pulmonary infections could potentially obscure the underlying malignancy.

IMA, defined as a distinct histopathological subtype of lung adenocarcinoma, predominantly occurs in nonsmoking females with a predilection for lower lung lobes.^10)^ Characterized by tumor-derived mucin infiltration into surrounding pulmonary parenchyma, nearly 100% of IMA cases exhibit ground-glass halo signs on chest CT imaging.^11)^ Mucin accumulation may also induce bronchial obstruction, leading to localized atelectasis and radiologically mimicking manifestations of KS.^12)^ Furthermore, IMA manifests pneumonia-like symptoms, including persistent cough, dyspnea, pleuritic pain, wheezing, and recurrent respiratory infections,^13)^ creating diagnostic overlap with KS-associated pneumonic presentations. Definitive diagnosis requires histopathological confirmation through CT-guided percutaneous biopsy, bronchoscopic sampling, transbronchial cryobiopsy,^14)^ or even surgical lung biopsy.

The potential association between KS and pulmonary mucinous adenocarcinoma remains unclear. However, previous evidence suggests that pre-existing pulmonary pathologies—such as chronic obstructive pulmonary disease and pulmonary fibrosis—are linked to an elevated risk of lung cancer, with chronic inflammatory pulmonary disorders potentially promoting carcinogenesis.^15)^ Notably, a population-based cohort study of over 3 million individuals demonstrated a significantly higher incidence of lung cancer in patients with bronchiectasis compared to non-bronchiectasis controls.^16)^ Mechanistically, ciliary dysfunction-induced chronic pulmonary inflammation in KS, combined with recurrent infections and subsequent parenchymal scarring, may drive carcinogenesis through persistent airway epithelial injury, triggering aberrant repair mechanisms and sustained hypercellular turnover that increase mutagenic susceptibility.^17,18)^ Furthermore, chronic airway inflammation promotes bronchial epithelium phenotypic changes, such as epithelial-to-mesenchymal transition, and modifies the tumor microenvironment. The resultant overproduction of tissue damage/repair-associated mediators (e.g., reactive oxygen species) triggers DNA damage and genomic instability, which collectively fuel lung carcinogenesis.^19,20)^ Infection-induced airway injury may act as a critical juncture promoting airway remodeling, apoptosis resistance, and angiogenesis, ultimately leading to the establishment of a pro-tumorigenic microenvironment in susceptible individuals.^19)^ However, further multicentric studies integrating ciliary genomics, inflammatory biomarkers, and longitudinal cancer registries are required to elucidate this potential etiological nexus.

KRAS mutations were detected in the tumor. Multiple studies suggest that the mutation may be associated with poor prognosis in lung cancer.^21,22)^ Notably, in patients with KRAS mutations, the KRAS-Akt pathway might facilitate the motility of neoplastic cells during the early period of carcinogenesis in lung adenocarcinomas and may contribute to their noninvasive expansion along the alveolar septa, leading to tumor cell spreading along the alveolar surfaces. This may explain why IMA exhibits pneumonia-like imaging features.^23)^ In summary, the clinical manifestations induced by these two gene mutations may overlap with those of KS syndrome, potentially complicating disease diagnosis and management.

In this case, the patient’s chronic mild cough and sputum production, combined with extensive inflammatory changes on chest CT, initially complicated the diagnostic process. The initial working diagnosis centered on pneumonia; however, negative results from bacterial cultures, fungal assays, and autoantibody panels preliminarily excluded both infectious etiologies and autoimmune-mediated pneumonitis. Malignancy remained a persistent consideration in the clinical differential diagnosis. The definitive diagnosis was only established after the tumor progressed to a solid mass amenable to percutaneous biopsy, despite a prior negative result from bronchoscopic sampling. This diagnostic delay highlights the challenges in distinguishing neoplastic from inflammatory processes in KS-associated lung disease. In addition, the surgical intervention was further complicated by the patient’s situs inversus, necessitating more meticulous anatomical orientation by the surgical team.

## Conclusions

This represents the first documented case of KS coexisting with pulmonary IMA, to some extent expanding the clinical spectrum of ciliopathy-associated lung malignancies. It provides a new perspective for the diagnosis of pulmonary mucinous adenocarcinoma in KS patients: For visceral situs inversus patients presenting with pulmonary symptoms, chest CT revealing diffuse inflammatory-like changes should raise suspicion not only for pneumonia but also for mucinous adenocarcinoma, particularly when accompanied by suspicious mass lesions. This suggests that clinicians and radiologists should consider the possibility of concurrent mucinous adenocarcinoma when dealing with KS patients.

## Declarations

### Availability of data and materials

The datasets used and/or analyzed during the current study are available from the corresponding author upon reasonable request.
